# Latest developments on the mechanism of action of membrane disrupting peptides

**DOI:** 10.52601/bpr.2021.200037

**Published:** 2021-06-30

**Authors:** Sara Pandidan, Adam Mechler

**Affiliations:** 1 La Trobe Institute for Molecular Science, La Trobe University, Melbourne, Australia

**Keywords:** Antimicrobial peptides (AMPs), Membrane disrupting peptides, Empirical models, Thermodynamic models

## Abstract

Antimicrobial peptides (AMPs) are integral components of the innate immune defence system of all complex organisms including plants, insects, and mammals. They have wide range of antibacterial, antifungal, antiviral, and even anticancer activities, therefore AMPs are attractive candidates for developing novel therapeutic approaches. Cationic α-helical membrane disrupting peptides are perhaps the most widely studied subclass of AMPs due to their common fundamental characteristics that allow for detailed structure-function analysis and therefore offer a promising solution to the threat of multidrug resistant strains of bacteria. The majority of the studies of AMP activity focused on the biological and biophysical aspects of membrane disruption; the understanding of the molecular mechanism of action from the physicochemical point of view forms a relatively small subfield. This review will provide an overview of these works, focusing on the empirical and thermodynamic models of AMP action.

## INTRODUCTION

The emergence of bacterial resistance to conventional antibiotics is one of the most significant international health issues of our time that requires an urgent solution (Cohen 1992; Livermore 2004). At the least, unnecessary antibiotic prescriptions should be eliminated to preserve the efficiency of the drugs that we have, however new antibiotics are also urgently required (Livermore 2004; Zumla and Grange 2001). It is critical that these new antimicrobial agents should kill or inhibit growth of pathogens quickly before allowing them to mutate and develop resistance. Previously, pharmaceutical industry had exacting requirements towards new drugs that focused attention on synthetic small molecules. However, the looming emergency broadened the scope: any working solution is acceptable when our modern way of life is under threat.

One of the potential alternatives to traditional antibiotics is the use of antimicrobial peptides (AMPs), as they can work quickly, efficiently, and have wide ranging activity (Hancock and Sahl 2006). These naturally occurring AMPs represent one of the initial and most effective forms of innate immune defence in all multicellular organisms (and have their equivalents in weapons of inter-bacteria warfare) (Diamond *et al*. 2009; Pasupuleti *et al*. 2012; Radek and Gallo 2007). AMPs are active against both Gram-positive and Gram-negative bacteria, while some of them show anticancer and antiviral activities as well (Hancock and Diamond 2000; Mangoni and Shai 2009) and they may also have activity against parasites and fungi; they are usually released at the time of hazardous situations such as facing the risk of a hunter’s attack (Conlon and Sonnevend 2010; Steiner *et al*. 1981; Zasloff 1987, 2002). AMPs typically contain 12 to 50 amino acids however with substantial differences in their sequence (Zasloff 2002). Hence, since their discovery, they were seen as a potential solution for the global health threat of antibiotic resistance (Dubos 1939; Steiner *et al*. 1981). Since they have evolved for the purpose of killing pathogens with high efficiency and since their mechanism of action fundamentally differs from conventional antibiotics, AMPs are less likely to give rise to AMP-resistant bacterial strains (Hoskin and Ramamoorthy 2008; Klotman and Chang 2006; Parisien *et al*. 2008).

**Figure 1:**
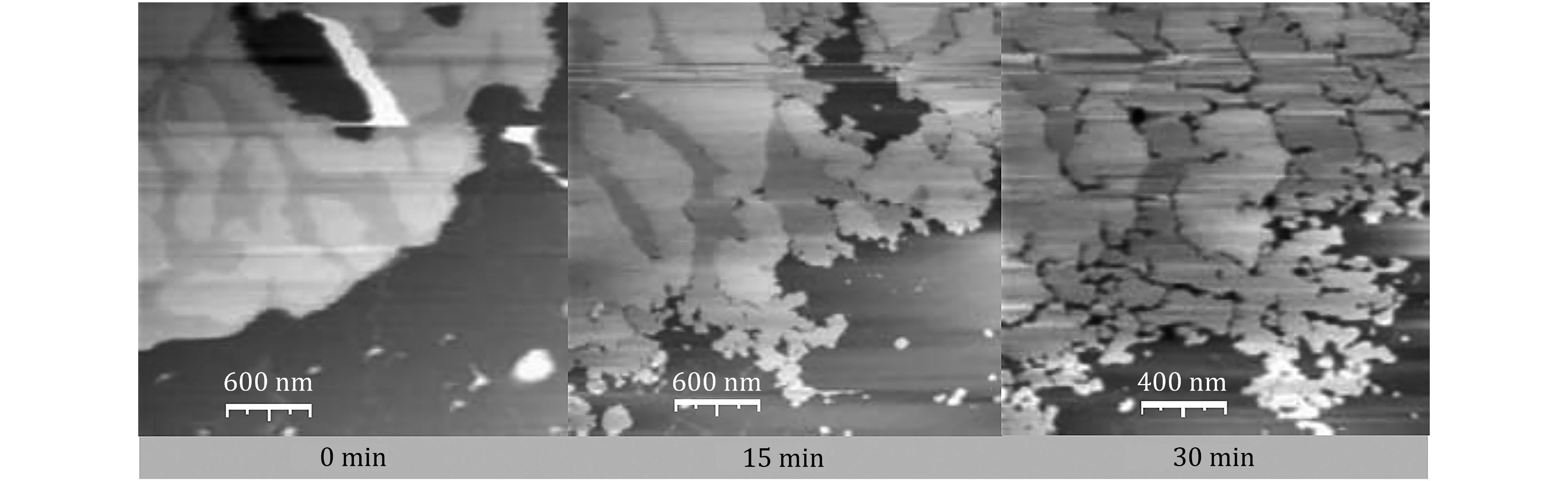
AFM images of interaction of pore former peptide melittin with DMPC:DMPG (4:1) model membrane show crack formation. Reproduced with permission from Springer Nature (Lee *et al*. 2009)

As the number of known AMPs expanded, distinct subclasses were identified based on their structure and/or way of action. Perhaps the most intensively studied subclass of AMPs is that of the α-helical cationic membrane disrupting peptides as they do not only have a unique mechanism but also exhibit common fundamental characteristics (Powers and Hancock 2003; Sato and Feix 2006). More than 100 AMPs have been identified to have α-helical structure, consisting 12 to 40 residues and may have a central “hinge” region (Pukala *et al*. 2004; Vouille *et al*. 1997). Specificity and selectivity of these AMPs is based on the differences between microbial and host cell membrane structures (Kabelka and Vacha 2015). Membrane disruption is suggested to proceed either through a surface acting mechanism that dissolves the membranes (Gazit *et al*. 1996; Shai 2002; Steiner *et al*. 1988) or discrete transmembrane pore formation (Huang 2000; Ludtke *et al*. 1996; Matsuzaki *et al*. 1998; Yang *et al*. 2001), either of which subsequently leads to cell death by upsetting osmotic balance (Huang *et al*. 2010). There are indications of more complex mechanistic pathways, as *e.g*. pore forming mechanism may take unexpected forms as seen in AFM image of crack formation upon addition of melittin on DMPC:DMPG (4:1) membrane (Fig. 1) (Lee *et al*. 2009). Membrane disruption is proven for all peptides of this class; however, the exact molecular mechanism of action leading to membrane disruption is still debated (Fuertes *et al*. 2011; Karal *et al*. 2015; Nguyen *et al*. 2009).

Membrane disrupting AMPs offer a unique solution to the treatment of multidrug resistant strains of bacteria (Fox 2013; Uhlig *et al*. 2014; Zhang *et al*. 2015). However, there are fundamental impediments towards turning AMPs into human pharmaceutics (Darveau *et al*. 1991; Uhlig *et al*. 2014). Mammalian AMPs are too large for cost effective synthesis but, given that they are antimicrobials, are not feasible to produce with microbiological methods (Dutta and Das 2016). Small AMPs originate from insects and amphibians, and, while specific and selective in their hosts, they are cytotoxic in humans (Slocinska *et al*. 2008). Hence small AMPs have to be modified for human use (Uhlig *et al*. 2014). That however presupposes a good understanding of the molecular interactions driving AMP activity, of which hypotheses exist but do not fully explain the observed level of specificity and selectivity of wild type AMPs (Shai and Oren 2001).

Attempts to modify AMPs for human use started from AMPs that exhibited some degree of specificity and selectivity. The mutations focused on improving activity by altering overall physicochemical characteristics of the peptides: helicity, hydrophobicity and cationic charge (Brogden 2005). With almost every attempt, the antibacterial activity of the peptides was increased, an optimum was found; however, the synthetic AMPs became more haemolytic and/or lost most of their specificity and selectivity compared to the wild type originals (Dathe *et al*. 2001; Dathe and Wieprecht 1999). Synthetic AMPs designed without a natural model also used the assumed physicochemical design motifs, but these artificial AMPs have not delivered the expected breakthrough: they require much higher concentration to perform the expected membrane disrupting behaviour (Yang *et al*. 2008), and they might even follow a different mechanism of action (Khara *et al*. 2014), although there are some encouraging results (Speck *et al*. 2014). Nevertheless, the data suggest that the real activity motifs of the AMPs should be sought at the residue level.

This situation is compounded by the inconsistency between biophysical studies that often disagree in the effect of a particular mutation, or even the overall mechanism of action (Guha *et al*. 2019; Pandidan and Mechler 2019). This variability of the data can be traced back to the lack of consensus on the desirable conditions and lipid compositions for membrane disruption studies, and hence activities are compared under vastly different conditions and thus extrapolating them to actual physiological environments and conditions is doomed to failure.

There is a pressing need to develop a residue level understanding of the drivers and controls of AMP action, that is, the physical chemistry aspects of the process. Most AMP studies focused on the phenomenological, biological and biophysical aspects of membrane disruption; comparably little attention was paid to the physical chemistry of AMP activity. Therefore, the focus of this review is the underlying mechanism: the often still hypothetical series of molecular interactions and processes leading to the membrane disrupting outcome.

## EMPIRICAL MODELS OF MEMBRANE DISRUPTING MECHANISM

While the exact mechanism of action of a particular membrane disrupting AMP is rarely known, and in many cases still debated (Hale and Hancock 2007; Hwang *et al*. 1998), the two distinct classes of membrane permeabilization mechanisms, that is, surface and pore forming action are widely accepted based on available data and the structure of AMPs (Bahar and Ren 2013; Brogden 2005; Wimley 2010). Transmembrane pore formation is divided into two different pore geometries known as barrel-stave and toroidal pores, and surface acting mechanism also has two subclasses, described by the carpet and the detergent models (Brogden 2005; Epand 2016; Kumar *et al*. 2018; Wimley 2010). It should be emphasized that all these proposed models classify peptide-membrane interaction via the final product of the disruption process while mostly neglecting to describe the mechanistic pathway leading to these outcomes.

The common ground of these models is that the cell killing activity is exerted via perturbation of the membrane and breaching its integrity (Ganz and Lehrer 1995). The classification relies on overall biophysical peptide characteristics (such as charge, hydrophobicity, amphipathicity, size and solubility) to predict which mechanism would a particular peptide follow (Chen *et al*. 2007; Dathe and Wieprecht 1999; Tossi *et al*. 2000). AMPs are unstructured in aqueous solution and adopt a typically amphipathic α-helical structure in the presence of lipid membrane; this amphipathic structure is essential for their affinity interactions with lipid membranes and is seen as the main reason why these AMPs are positioned at the interface between the hydrophobic membrane core and the aqueous medium and/or aggregate into transmembrane bundles (Lee *et al*. 2014; Sani and Separovic 2016). Furthermore, the preferred mechanism shows a weak correlation to the peptide length (Wimley 2010). It is explained with match or mismatch between membrane thickness and the length of the helix, arguing that both short (<3 nm helical length) and too long (>7 nm) peptides have difficulty forming pores through the membrane. 4-nm long helices, that corresponds to approximately 20–30 amino acid residues in the α-helix, are seen as optimal to span a lipid membrane of similar thickness by forming pores (Lee *et al*. 2004). These characteristics are sufficient to establish what might be described as phenomenological membrane disruption models; in the followings these will be described first, before discussing the thermodynamic aspects of AMP action.

### Barrel-stave pore model

**Figure 2:**
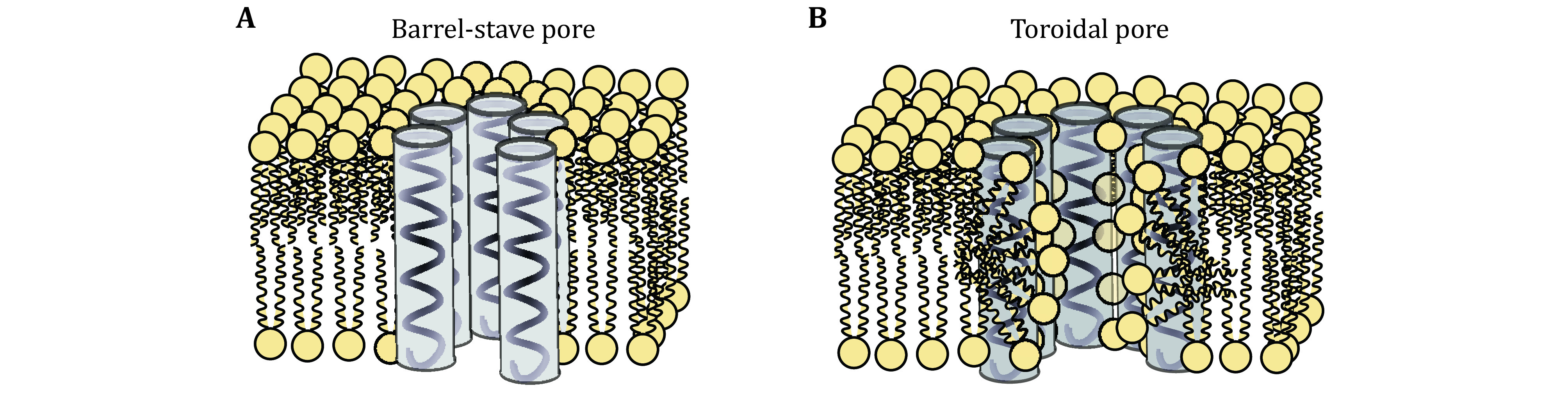
The two main geometries of pore forming action. **A** Barrel-stave pore. **B** Toroidal pore. In the schematic representation of the lipids the head groups are shown in yellow and the peptides are shown fully helical

Barrel-stave pore formation as a means of membrane disruption was first proposed by analysis of steady-state fluctuations of single-pore conductance in 1974 to explain alamethicin activity in black lipid membranes (Baumann and Mueller 1974; Boheim 1974). In this hypothetical mechanism first the peptide monomers bind to the membrane and form an α-helical structure. Then the peptides aggregate on the surface to form a permanent bundle with a narrow hydrophilic central hole, essentially an ion-channel that consecutively penetrates the core region of membrane, without significant perturbation of the lipid molecules in the way that their hydrophobic surfaces interact with the lipid core and their hydrophilic surfaces form the interior region of an aqueous pore (Fig. 2A) (Boheim 1974; Boheim and Benz 1978; Boheim *et al*. 1983; Cafiso 1994; Estep *et al*. 1978; Mak and Webb 1995; Oren and Shai 1998).

The logic of the model necessitates that hydrophobic interaction is the main driving force in this mode of action (Oren and Shai 1998). A more generic model of the barrel-stave mechanism involves four main stages: (1) initial electrostatic binding of the helical peptide most likely in monomeric form to the membrane surface, (2) membrane insertion of the hydrophobic region of the bound peptides to a depth that varies based on the membrane outer leaflet hydrophobicity, (3) after a threshold concentration, the bound peptide monomers self-aggregate and may insert deeper into the membrane core region, (4) the peptide hydrophobic faces align to the hydrophobic lipid core region, while their hydrophilic regions form the interior part of a water-filled pore (Park *et al*. 2011). The pore size and the pore former oligomeric state would be then determined by the distribution of the cross sectional amphiphilicity of the helix: the narrower the hydrophilic face, the smaller the pore. However, there is not any direct or indirect evidence for the existence of these intermediate stages.

It should be noted that the tightly packed peptide bundle as required for this mode of action is only feasible for very weakly charged peptides that can be tightly packed; else the Coulombic repulsion would destabilize the pore and/or inhibit its formation (Ludtke *et al*. 1996; Yang *et al*. 2001; Zemel *et al*. 2003). Consistently, barrel-stave pore formation might only be possible for a small number of ideally proportioned peptides; this unique pore formation in alamethicin is well studied and confirmed, but alamethicin is the only known peptide following this model (Lee *et al*. 2004; Yang *et al*. 2001). It should be noted that based on geometrical consideration it was assumed that alamethicin pore would be formed by a fixed number of monomers (Hall 1975), however there is evidence that the pore size can increase with monomers joining the aggregate, detected from increasing ion conductance (Matsubara *et al*. 1996; Sansom 1991).

### Toroidal pore model

Toroidal pore or wormhole mechanism was first proposed in 1996 to describe magainin-induced pores (Ludtke *et al*. 1996). In the toroidal pore mechanism, AMP insertion into the membrane lead to asymmetric tension that forms pores by induced surface bending in membrane leaflets once a critical threshold concentration is reached, bridging the two leaflets in a sharp curve so that the water core is lined by both the inserted peptides and the lipid head groups (Fig. 2B) (Ludtke *et al*. 1996; Yeaman and Yount 2003). By disruption of the bilayer curvature and forming a torus, the inserted peptides would cause permeabilization, or disintegration of the membrane (Marsh and Goode 2007; He *et al*. 1995). The main difference between barrel-stave and toroidal model is that in the toroidal pore model, peptides are always associated with the lipid head groups even when they reside in the pore (Sengupta *et al*. 2008; Yang *et al*. 2001). Thus, toroidal pore formation is caused by peptides that do not insert into the membrane core, as even in the torus they are essentially on the membrane surface. Hence it can be seen as an extreme case of surface action as it will be detailed below.

A peculiar aspect of the toroidal pore formation that many, but not all, peptides assumed to follow this mode of action do only disrupt the membrane at a threshold concentration (Cudic and Otvos Jr 2002). Yet there is a contradiction: this model also suggests that the torus itself is not necessarily the lowest energy state for the peptide in the membrane; hence the formation of stable toroidal pore is highly dependent on a critical peptide-lipid ratio, and increasing peptide concentration could actually lessen the pore stability because of higher electrostatic repulsion between the peptides (Brogden 2005). Furthermore, while this mode of disruption is ascribed to many AMPs, in spite of the supposedly large size of the pore compared to a barrel-stave bundle, toroidal pores have not been observed with microscopy methods; indeed in case of melittin, a markedly different pore geometry was imaged (Fig. 1).

### Carpet model

**Figure 3:**
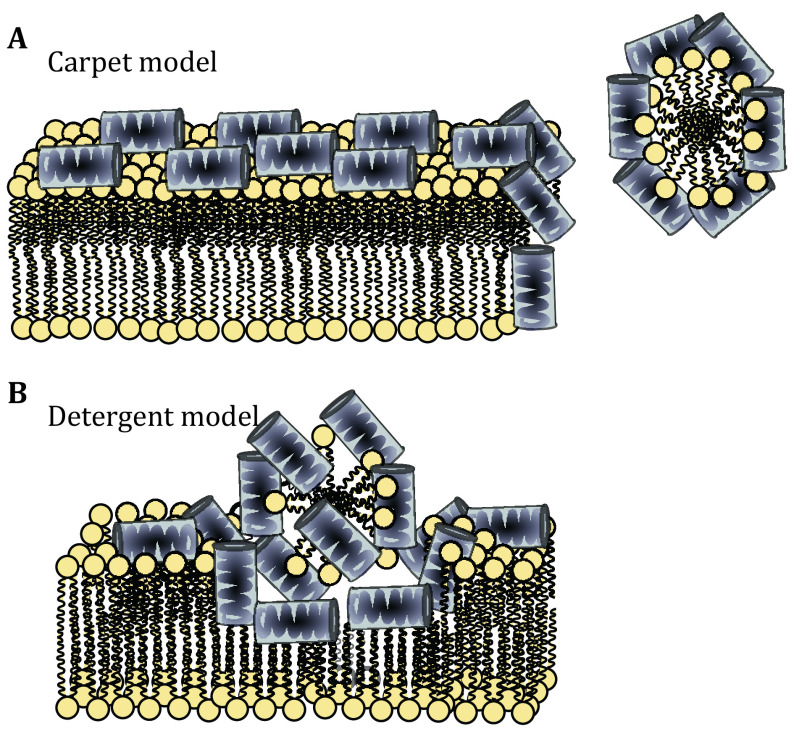
Surface action of antimicrobial peptides. **A** Carpet model. **B** Detergent model. In the schematic representation of the lipids head groups are shown in yellow and the peptides are shown fully helical

The so called carpet model was first proposed by Pouny *et al*. in 1992 for explaining the mechanism of action of dermaseptin (Pouny *et al*. 1992). Carpet mechanism is described in four steps: (1) Peptide monomers adhere parallel to the membrane surface; it is assumed that the main force of attachment in this mechanism is electrostatic attraction between the negative charge of bacteria membrane surface and positive charge of the peptides (Mani *et al*. 2004; Shahmiri *et al*. 2015). (2) Peptide obtains helical fold on the surface of the membrane in a way that the positive charges of amino acids interact with the negatively charged lipid headgroups or water molecules, covering the membrane surface in a carpet-like fashion. (3) Rotation of peptide leading to reorientation of the hydrophobic peptide residues toward the hydrophobic membrane core region. (4) Finally breaking down the membrane by disrupting the bilayer curvature and forming micelles (Fig. 3A) (Oren and Shai 1998). The actual mechanism of the last step is not well described even in the hypothesis, it was suggested that formation of transients pores in the membrane enabling the passage of low molecular weight molecules could be the initial stage before complete membrane collapse (Oren and Shai 1998). The parallel orientation of the peptide on the membrane surface during the whole process may change the membrane fluidity by displacing phospholipids and that could distort and destabilize the phospholipid packing (Powers and Hancock 2003; Shai and Oren 2001). However, that would be a gradual process whereas it is well documented that in this mechanism, as in the toroidal pore model, there is also a critical peptide threshold concentration (Fernandez *et al*. 2012; Gazit *et al*. 1995; Shai 1999; Shai and Oren 2001).

The existence of the threshold can be explained with an adjustment to the above model, noting that the presence of the peptides in the top membrane leaflet alters surface tension and thus when the occupancy of surface bound state is high enough the torque of the asymmetric lateral tension between the two membrane leaflets overcomes the hydrophobic force that holds the membrane intact and thus the membrane breaks up (Mechler *et al*. 2007, 2009; Shahmiri *et al*. 2015).

It is important to note, however, that there is little to no difference in the reasoning between the toroidal pore and carpet model, yet a substantially different outcome is proposed. Consistently it was suggested that carpet mechanism could be an extreme form of the toroidal pore mechanism (Dathe and Wieprecht 1999).

### Detergent model

Detergent model may be seen as the extended version of the carpet model of AMP action. Some authors combine these two models, suggesting that comprehensive breakdown of the membrane integrity includes membrane fragmentation into micelles (Wimley and Hristova 2011). Others differentiate between the two models based on whether or not the peptide-induced leakage efficiency depends on the size of the entrapped solutes (Ostolaza *et al*. 1993). The initial interaction of peptide in detergent model is similar to the carpet model, though without a threshold: even at lower concentration the peptide is causing some dye leakage which is caused by the “budding” of mixed micelles that at higher concentration leads to direct dissolution of the membrane and thus cell death (Fig. 3B) (Goyal and Mattoo 2016; Kumar *et al*. 2018). It should be noted that often the term "detergent effect" has been cited to explain the catastrophic membrane integrity loss at high concentration of peptides in other mechanisms (Bechinger and Lohner 2006; Hristova *et al*. 1997; Ostolaza *et al*. 1993).

### Critique of the phenomenological models

It is important to note that most of the known AMPs exhibit anomalies in the expected behaviour based on either of these models (Dathe and Wieprecht 1999; Lehrer *et al*. 1989; Zasloff 2002). Some studies cast doubt on the very existence, or at least the importance of the four different membrane breaching pathways, arguing that allowing sufficient time all AMPs may fragment the membrane bilayer into micelle structures, regardless of their preferred mechanism (Hoskin and Ramamoorthy 2008; Shai 2002; Zasloff 2002). Other than for the barrel-stave mechanism of alamethicin there is scarce evidence in support of either of these mechanistic pathways, mainly due to the lack of suitable methodology. The peptides themselves, and even the peptide induced membrane structures are small, dynamic and highly environment dependent, thwarting microscopic observation and most biophysical and physicochemical methods that could be used to characterize the molecular processes. Furthermore, the models are limited to molecule level biophysical characteristics to predict the membrane interactions, such as overall charge, hydrophobicity, amphiphilicity and length; however, these parameters cannot fully explain the specificity and selectivity of the action of these peptides observed in microbiological studies (Brogden 2005). Attempts to identify drivers and motifs of activity at the residue level delivered mixed results, given that mutations of the peptides may change multiple properties and therefore randomization and alanine screen methods that are frequently used in medicinal chemistry necessarily fail on AMPs (Cantisani *et al*. 2014; Cunningham and Wells 1989; Hawkins *et al*. 1993). The failure of linking chemical structure to the empirical models has been thus far the greatest impediment towards rational design of AMPs for human therapeutics.

## THERMODYNAMIC MODELS

Thermodynamic models of peptide–membrane interactions rely on the chemical parameters of the peptides and the lipid membrane, and a morphological model of the interaction (the term “structural” is often used but it implies a static assembly whereas these models assume dynamic assemblies) (Seelig 2004). The process is described as a chain of equilibria, where each step of the mechanism is a distinct thermodynamic state, defined by the free energy of the specific peptide-membrane interactions and entropic factors. The occupancy of these states is defined by the Boltzmann distribution. Assuming that the states proceed from surface attachment to the final broken-up membrane in the order of increasing free energy, the occupancy of the states must have a clear concentration dependency, explaining the existence of thresholds in the process. The energy of each state can be calculated based on a model, or potentially measured with calorimetric methods. Even though thermodynamical modelling offers the means of gleaning much better insights to the dynamic process than the common structural approaches, there is only scarce data in the literature.

From the thermodynamic point of view, the membrane might be described as a platform where the peptides occupy various states, depending on how they interact with the lipid molecules and each other. This is a simplification, as the presence of the peptide alters the membrane structure as well, but a simplification that makes it possible to construct various sequences of states, *i.e*. mechanistic pathways for the peptides to follow. Sources mostly agree that the initial stages of peptide–membrane interaction can be divided into three thermodynamic steps: unstructured solution state, unstructured surface binding due to electrostatic attraction, and conformation change into the α-helical membrane disrupting fold (Ennaceur *et al*. 2009; Hall *et al*. 2014; Hirst *et al*. 2013; Jacobs and White 1989; Ningsih *et al*. 2012; Seelig 2004). Yet, even this simple process is called into question: it was shown recently that phenylalanine residues play a key role in membrane attachment, and removal of these residues eliminates activity, even if the charge is not altered; this was demonstrated for the surface acting aurein1.2 but it is feasible to assume broader validity of the observations (Shahmiri *et al*. 2017). It was also shown that the C-terminal amidation plays a necessary and unique role: without amidation many AMPs are inactive, which was explained before with charge effects (Huang *et al*. 2010; Strandberg *et al*. 2007; Strøm *et al*. 2002). However, it was recently demonstrated, also for aurein1.2, that methylamidation deactivates the peptide as well, even though it delivers the same cationic charge increase (by eliminating a negative charge) and structural effects (through hydrogen bonding) as simple amidation (Shahmiri and Mechler 2020). These results question the role of charge in the disruption process, suggesting that there can be other factors that are hitherto unaccounted for. Further uncertainty surrounds the steps leading from surface binding to membrane disruption, as these are difficult to analyse due to the dynamic nature of the process, and that may also involve nucleation at membrane defects (Karatekin *et al*. 2003; Melikov *et al*. 2001; Weaver and Chizmadzhev 1996).

### Two and three stage pore forming models

From a purely energetic point of view the main reason for the formation of stable pores would be that the pore is the lowest energy state for the peptides to occupy in the membrane (Afonin *et al*. 2008; Huang 2009; Huang *et al*. 2004; Lazaridis *et al*. 2013). This is the case when the AMP helix is mostly hydrophobic with a narrow hydrophilic face: such peptides may form permanent small channels of the barrel-stave geometry because only the central part of the pore would be water-loving and all the hydrophobic residues can favourably interact with the membrane core and each other (Brogden 2005). It should be noted that this assumption may be incorrect, as a free energy calculation study suggested that the thermodynamic mechanism for the formation of both types of pores (*i.e*. barrel-stave and toroidal) are the same and the only difference is the free energy of the pore itself (Huang 2009). Nevertheless, the simplest energetic model of membrane disruption contains two states for peptide-membrane interaction, the S-state for the surface bound state of peptide and the I-state for the membrane inserted monomeric state (Huang 2000, 2006). This does not account for the different, supposedly lower energy state of the pore forming aggregate therefore a third state of pore expansion known as E-state was proposed for the formation of a pore-like structure by in-membrane aggregation resulting from continued insertion of peptide (Rakowska *et al*. 2013). Such simplistic models, however, fail to deliver results of acceptable predictive validity.

### Flip-flop pore model

The above assumption of a lower energy pore state might not be valid for highly amphiphilic peptides. For these the membrane insertion is a higher free energy state than the surface bound state, since the polar face of the helix may remain hydrated allowing water penetration into the membrane core, and the energy cost of breaking the physical bonds to the lipid headgroups is not fully recovered by the weak van der Waals type interactions in the membrane core. If only the energy difference is considered such peptide would not enter the membrane; however, in a dynamic system, the membrane inserted state of such peptides is sparsely populated due to the mixing entropy; the relative occupancies are described by the Boltzmann distribution. Thus a modification of the two state model can be introduced assuming that transient pores might be resulted from this higher energy membrane inserted state of the peptides, as they are flip-flopping in and out of the membrane, potentially also leading to transfer between the membrane leaflets (Kim *et al*. 2009; Ludtke *et al*. 1996; Matsuzaki 1999). This kind of two-state system was extensively studied for the peptide magainin2 secreted by African clawed frog *Xenopus laevis* (He *et al*. 1995; Karal *et al*. 2015; Ludtke *et al*. 1996; Papo and Shai 2003; Tamba and Yamazaki 2005, 2009). Association of monomeric peptide to the headgroup region of the outer leaflet of the membrane is defined as B_ex_ state (Hirsh *et al*. 1996; Tamba and Yamazaki 2009). Increasing the concentration leads to more and more peptides "dipping" into the membrane, carrying with them some lipid molecules from the top leaflet and allowing water to penetrate the membrane at the hydrophilic side of the peptide. This is called the P (pore) state; when involving multiple peptides this could lead to the formation of dynamic, transient toroidal pores (Ludtke *et al*. 1996). Since these pores are not stable, they are expected to close once excess peptide is transferred to the inner leaflet through the curved membrane surface of the torus and an equilibrium develops due to the even distribution of monomers at both sides of the membrane (Ludtke *et al*. 1995, 1996; Matsuzaki *et al*. 1995). This model does not assume any peptide aggregation in the membrane, which was confirmed with NMR measurements (Hirsh *et al*. 1996). Dual polarisation interferometry studies of magainin2 on model membranes suggested that the membrane can recover when the excess peptide is removed from the solution, confirming the existence of a dynamic equilibrium, but the disruption can also proceed towards complete membrane lysis if the peptide concentration is increased (Hall *et al*. 2014).

### Membrane penetrating fissure model

**Figure 4:**
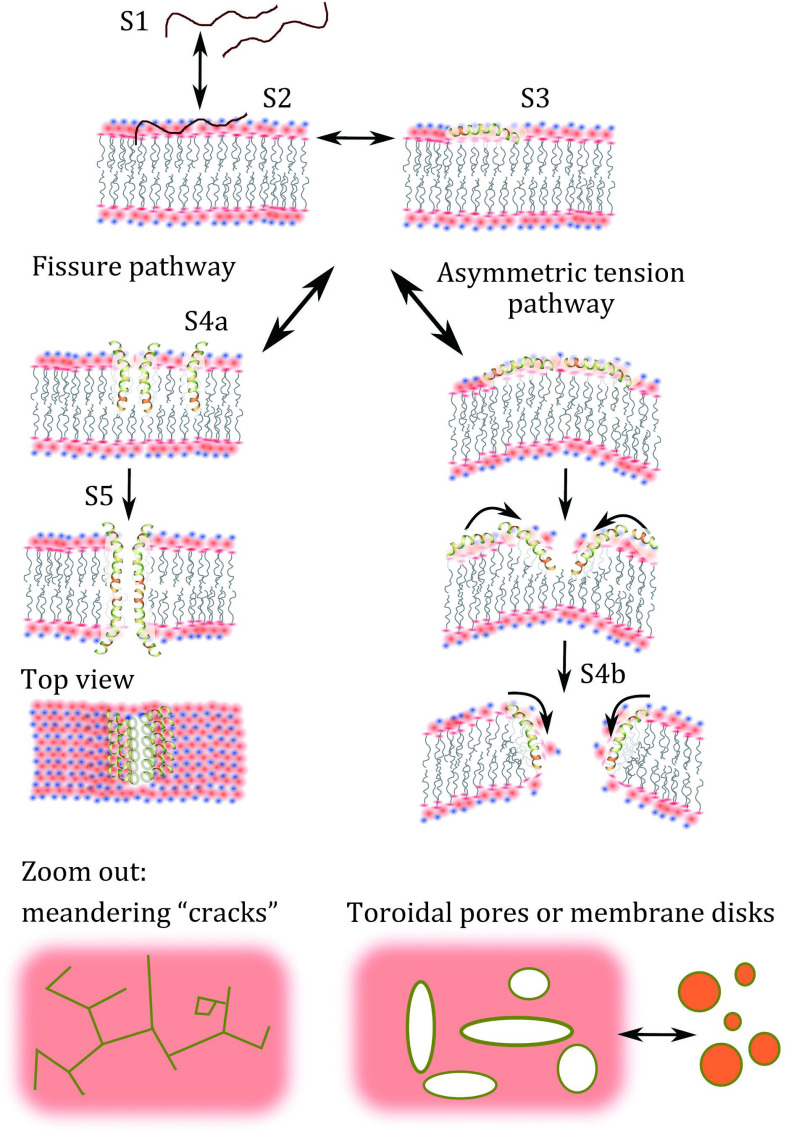
Schematic hypothetical pathways of fissure and asymmetric tension for melittin pore forming mechanism. S1, S2 and S3 are solvated, adsorbed and helical state of peptide, respectively, leading to S4a, S4b as linear “crack” assembly or toroidal pore formation, respectively. Reproduced from Pandidan and Mechler (2019) under Creative Commons license 4.0 (http://creativecommons.org/licenses/by/4.0/)

The most complex semi-empirical thermodynamic model of membrane disruption to date is the fissure model. Developed to explain the mechanism of action of melittin, it is supported by real time microscopy evidence as well as QCM viscoelastic fingerprinting (Pandidan and Mechler 2019). When parts of the peptide are highly charged but it also contains a sufficiently hydrophobic helical segment, a situation might arise where the peptide can insert in the membrane partially with the charged segment firmly attached to the lipid headgroups and the helix penetrating the membrane core. In the fissure model the peptides thus partially insert in monomeric form into the top membrane leaflet; given the polar-to-apolar ratio of the inserted helix is ~1:3, the insertion is not costly in energy terms compared to the surface bound state. The schematic representation of the fissure pathway is shown in Fig. 4 (Pandidan and Mechler 2019).

The hydrophilic face of the inserted helix however allows in-membrane aggregation of the peptide; with essentially ¼ of the helix being hydrophilic, the preferred aggregation geometry is a linear fissure. This fissure may expand as more monomers join the aggregate, leading to substantial water penetration and hence unbalancing the bottom membrane leaflet, exposing the hydrophobic core; energetic stability can be achieved by flip-flopping peptides through the fissure to the bottom leaflet side, completing the transmembrane fissure. Spreading of the fissures may break up the membrane to small island or form peptide-stabilized nano discs. There is microscopy evidence of this process (Lee *et al*. 2009).

### Asymmetric tension model

If the amphiphilic cross section of the helix approaches an even distribution along its full length, it is too long, or in case of specific (charge or hydrogen bonding) interactions between the polar face of the amphiphilic helix and the lipid headgroups, as is the case of charged bacterial membranes, the energy cost of membrane penetration is too high: the occupancy of the membrane inserted state is negligible, the peptides remain on the surface (Shahmiri *et al*. 2015, 2016). Continuing peptide adsorption can have various effects on the membrane: it may dilute the membrane and changes fluidity, leading to instability (Shahmiri *et al*. 2015, 2016); if that does not proceed to the flip-flop mechanism for reasons outlined above, it leads to a gradual increase in the membrane tension in the top leaflet, while the bottom leaflet is unaffected. The peptides may remain monomeric (Shai and Oren 2001) or may aggregate and partially insert into the top leaflet (Shahmiri *et al*. 2015). The resulting surface tension difference introduces a spontaneous curvature in the membrane, a torque that, when reaching the lateral membrane tension, may cause blistering and/or eventual catastrophic breakdown of the membrane (Hartmann *et al*. 2010; Shahmiri *et al*. 2015). One possible pathway is shown in Fig. 4 (right side). The energetic model in this case is very simple, given that the peptide remains in essentially one state throughout, the surface adsorbed state, although for aggregation and partial penetration further intermediate states are introduced; however, the membrane disruption itself is described in terms of the resulting surface free energy changes of the membrane itself.

It is easy to see that under specific conditions the asymmetric tension may not proceed to complete membrane breakdown but lead to the formation of toroidal type pores (Pandidan and Mechler 2019) as also described in the pore expansion model (Chen *et al*. 2002, 2003). Hence this model is distinct from the flip-flop model that describes transient, unstable pores.

## SUMMARY

The models outlined in this review are, to a large part, hypothetical. The available evidence supports certain aspects of the models, however none of them has been proven unequivocally. The field has taken enormous strides in the 1990s and early 2000s in understanding AMP action, however by now that initial momentum is spent without reaching the goal: development of AMPs into viable drug candidates for human therapeutics. This is the time to revisit the fundamentals and develop in-depth understanding of the molecular interactions leading to membrane interaction, to further develop and parametrize the thermodynamic models. Such knowledge will reveal design motifs and allow for rational design of membrane disrupting peptides for specific goals, to kill bacteria or fungi, eliminate cancerous cells, or disrupt the envelope of viruses.

## Conflict of interest

Sara Pandidan and Adam Mechler declare that they have no conflict of interest.
